# The Influence of Rheumatoid Arthritis on Higher Reoperation Rates over Time Following Lumbar Spinal Fusion—A Nationwide Cohort Study

**DOI:** 10.3390/jcm11102788

**Published:** 2022-05-16

**Authors:** Jin-Sung Park, Se-Jun Park, Jiwon Park, Gijun Shin, Jae-Young Hong

**Affiliations:** 1 Department of Orthopedics, Samsung Medical Center, School of Medicine, Sungkyunkwan University, 81 Irwon-ro, Gangnam-gu, Seoul 06351, Korea; paridot@hanmail.net (J.-S.P.); sejunos@gmail.com (S.-J.P.); 2 Department of Orthopedics, Korea University Ansan Hospital, 123 Jeokgeum-ro, Danwon-gu, Ansan-si 15355, Korea; jwpark506@gmail.com (J.P.); lemonsmile82@hanmail.net (G.S.)

**Keywords:** lumbar spinal fusion, rheumatoid arthritis, reoperation, risk

## Abstract

This study aimed to compare the rates of reoperation over time following first lumbar fusion in rheumatoid arthritis (RA) patients and non-RA patients. This study was conducted using Korean Health Insurance Review and Assessment (HIRA) data. We identified the RA group as 2239 patients who underwent their first lumbar fusion with RA and the control group as 11,195 patients without RA. This reflects a ratio of 1:5, and the participants were matched by sex, age, and index surgery date. The index dates were between 2012 and 2013. When comparing the rate of patients undergoing reoperation, the adjusted HR was 1.31 (95% CI: 1.10–1.6) in the RA group (*p* = 0.002). In terms of the three time intervals, the values in the time frames of <3 months and 3 months–1 year were not statistically significant. However, at 1 year post-surgery, there was a higher risk of reoperation in the RA group, as demonstrated by the Kaplan–Meier cumulative event analysis. This higher risk of reoperation continued to increase throughout 5 years of follow-up, after which it was stable until the last follow-up at 7 years. This population-based cohort study showed that the RA patients had a 1.31 times higher risk of reoperation following lumbar fusion than did the controls. This difference was more pronounced at 1 year post-surgery.

## 1. Introduction

Rheumatoid arthritis (RA) is an autoimmune disease affecting the synovial joints and involves the cervical spine in 43–88% of cases [1]. However, there have been few studies concentrating on the influence on the lumbar spine. RA is a systemic inflammatory disease with significant osteoarticular involvement, and knowledge of its effect on the lumbar spine has largely been limited to anecdotal evidence from small patient populations.

A few studies have indicated that patients with RA undergoing lumbar spinal surgery have higher rates of complications, and that the overall clinical outcome might be worse than for patients without RA [2,3]. Similarly, with the increasing global burden of RA and findings suggesting that cervical and lumbar spine lesions can co-occur in patients with RA, understanding the true impact of the disease in patients undergoing lumbar spine surgery is important [1,4].

There are multiple reasons for reoperation after lumbar spinal fusion, such as adjacent segment disease (ASD), implant problems, and wound-related complications. Few authors have attempted to identify the risk factors for postoperative ASD, and these researchers have reported a higher rate of revision surgeries in patients undergoing spinal fusion compared to the non-fusion group [5]. Some have concluded that the rate of wound- and implant-related complications is higher in patients with RA undergoing lumbar spine surgery [6]. All of these studies have used small patient cohorts and were restricted to a single institution. There have been no nationwide, population-based studies analyzing the reoperation rates in patients with RA undergoing lumbar spinal fusion. Population-based studies provide the distinct advantage of high statistical power and are less prone to bias, which commonly affects small case series.

Therefore, this study aimed to analyze the rates of reoperation in RA patients undergoing first lumbar spinal fusion compared to patients without RA using Korean Health Insurance Review and Assessment (HIRA) data. Furthermore, our population-based cohort study compared the rates of reoperation between three subgroups based on the time between index surgery and reoperation.

## 2. Materials and Methods

### 2.1. Data Source

This study used a nationwide HIRA dataset. In South Korea, the government has implemented an obligatory National Health Insurance (NHI) system covering 97% of the population that allows patients to pay only about 30% of the total healthcare cost. The remaining 3% of the population, the lowest income households, has the Medical Aid Program that covers all medical expenses. Healthcare institutions submit claims for the total medical costs to the government. Therefore, the medical information of almost all patients in healthcare institutions is prospectively integrated into the HIRA claim database. This database includes extensive information on the diagnoses and comorbidity codes classified by the International Classification of Diseases, 10th revision (ICD-10); demographic characteristics; admission and ambulatory care; prescription records; and procedure codes

### 2.2. Study Design and Cohort

**Figure 1:**
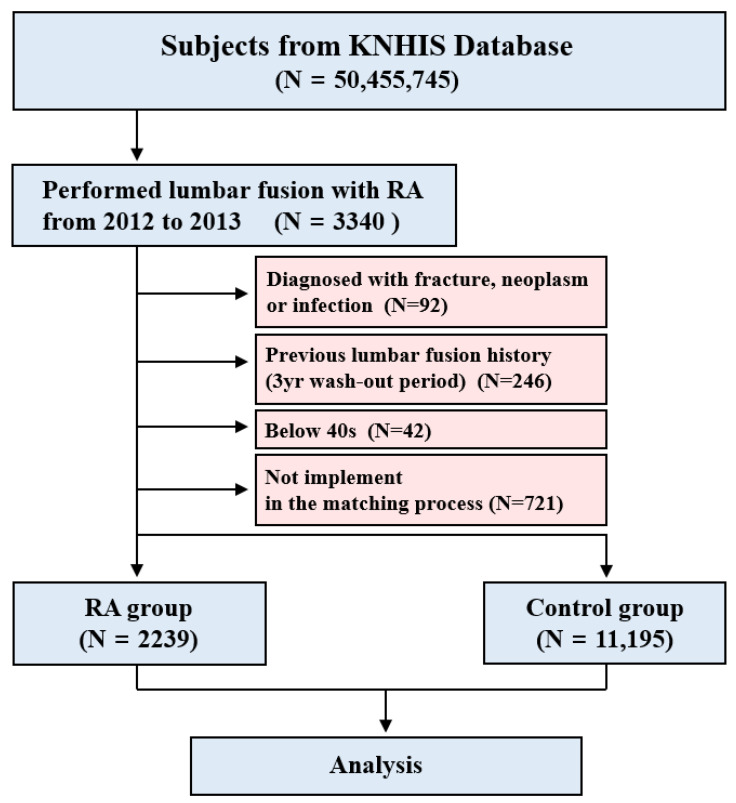
Flowchart of subjects from the Korean Health Insurance Review and Assessment Service (HIRA).

We filtered the ICD-10 codes to identify all the patients with RA who were included in the HIRA database. In the RA patient database with the ICD-10 codes (M05 and M06), we identified RA participants who underwent their first lumbar fusion (HIRA procedure code N0466, N0469, N1460, N1466, or N1469) between January 2012 and December 2013. Patients who underwent surgery for fracture, neoplasm, and infection were excluded. Patients who underwent any other lumbar surgery such as laminectomy, discectomy, or fusion (HIRA procedure code N1499, N1493, N1494, N1495, N1496, N0466, N0469, N1460, N1466, or N1469) before their first lumbar fusion were excluded. Patients under 40 years of age were excluded. Then, the RA subjects were matched by gender and age with control subjects in a 1:5 ratio. The patients in the control group were randomly sorted before being selected from a list to prevent bias. The RA group, without five control individuals who underwent their first lumbar fusion at the same time, was excluded in the matching process (*n* = 721). Then, we identified the frequency and type of lumbar reoperations for the RA and control groups (Figure 1).

Using the ICD-10 codes, the selected baseline comorbidities that could act as confounding factors included diabetes mellitus, depression, osteoporosis, Parkinson’s Disease, peripheral vascular disease, end stage renal disease, liver disease, hypertension, cerebrovascular disease, and chronic pulmonary disease [7]. Comorbidities were recognized in individuals who had three or more clinic visits for the disease as either a principal or secondary diagnosis. To increase diagnostic code validity, the suitability of prescription drugs was reviewed for the selected diseases.

**Ethical statement.** The study protocol was approved by the KNHIS Institutional Review Board. An informed consent exemption was granted by the board.

**Statistical analysis.** The Chi-square test was used to examine the different demographic variables and baseline comorbidities between the RA and control groups. The reoperation-free survival rate and differences between the two groups were evaluated using the Kaplan–Meier method and the log-rank test, respectively. Cox proportional hazards regression analysis was used to calculate the hazard ratio (HR) and 95% confidence interval (CI) for reoperation for the RA group compared with the control group. Reoperation rates were analyzed for three time intervals (0–90 postoperative days, 91–365 days, and 366 days to 7 years). The multivariate Cox proportional hazards model was used to identify the risk factors for reoperation and to determine the adjusted hazard ratio of the RA cohort. Statistical analyses were performed using SAS version 9.3 (SAS Institute Inc., Cary, NC, USA). *p* values less than 0.05 were considered statistically significant.

## 3. Results

### 3.1. Characteristics and Comorbidities between the Two Groups

The distributions of the demographic variables and chronic comorbidities in the RA and control group patients are shown in Table 1. The mean age of patients was 64.5 ± 8.8 years, and, because of the 1:5 age- and gender-stratified matching, this was comparable between the groups (*p* = 0.997). The age group was subdivided into four groups: 40–49 years, 50–59 years, 60–69 years, and ≥70 years. The number of male patients constituted 26.9% of the patients in the RA group and 27.1% patients in the control group (*p* = 0.876). When comparing the various comorbidities, we noticed a significantly higher rate of DM, depression, osteoporosis, peripheral vascular disease, liver disease, hypertension, and chronic pulmonary disease among patients in the RA group compared to the control group (*p* < 0.05).

### 3.2. Risk of Reoperation in RA Patients

When comparing the number of patients undergoing reoperation, 265 of 2239 patients in the RA group underwent revision surgery, accounting for 11.8% of the participants in this group. In the control group, 985 of 11,195 patients underwent reoperation, accounting for 8.8% of the participants in this group. The adjusted HR was 1.31 (95% CI: 1.10–1.6) in the RA group, which was statistically significant with a *p* value = 0.002. This suggests a higher rate of reoperation in the RA group compared to the controls (Table 2).

### 3.3. Risk of Reoperation in RA Patients with Progression over Time

**Figure 2:**
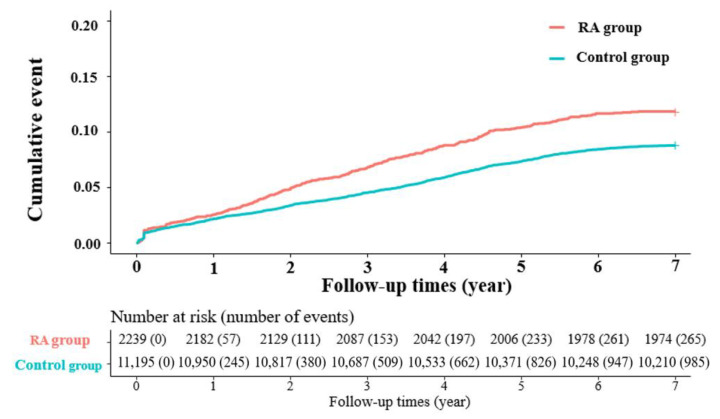
The Kaplan–Meier curves showed significantly higher cumulative reoperation rates in rheumatoid arthritis (RA) patients than in those in the non-RA control group.

We also tried to compare the rates of reoperation between the three subgroups based on the time between index and revision surgeries. The most remarkable difference between the RA group and the control group was observed in the >1 year time frame. There were 208 reoperations, account for 9.29% of participants in the RA group as compared to 740 reoperations, account for 6.61% of participants in the control group. The adjusted HR was 1.31 (95% CI: 1.11–1.57) with a *p* = 0.002, demonstrating a significantly higher rate of reoperation in the RA group after 12 months from the index surgery. The values in the time frames of <3 months and 3 months–1 year were not statistically significant (Table 3). Figure 2 shows the Kaplan–Meier curve for the cumulative incidence of reoperation of the RA and control groups during 7 years of follow-up. Among the RA group, there was a higher risk of reoperation, and the gap became more evident after 1 year (*p* < 0.0001). This gap widened after 1 year and reached its maximum at 5 years. This gap was maintained until the last follow-up at 7 years.

### 3.4. Subgroup Analysis of the Risk of Reoperation in RA Patients

A subgroup analysis was performed to estimate the rate of reoperation among the demographic factors and various comorbidities in the study. This was further analyzed among all patients undergoing reoperation and according to the three time frames previously described. There was a higher rate of reoperation in patients with depression overall. When extending this to the time frame analysis, this effect was more pronounced in the <3 months group with an adjusted HR of 2.37 (95% CI, 1.09–5.19) and a *p* = 0.003. This was also found in patients with osteoporosis, with an adjusted HR of 2.14 (95% CI, 1.15–4.02) and a *p* = 0.017. However, this finding was not significant in the individual analysis for the other time frames (Table 4).

## 4. Discussion

The current study has provided nationwide data regarding the comparison of reoperation rate in patients undergoing lumbar spine fusion based on RA as a comorbidity. There have been multiple previous reports describing abnormal radiological findings in the lumbar spine of patients with RA compared to normal patients [1,8]. Since the introduction of biological agents in the management of RA, the number of severe cervical lesions is expected to decrease with a main pathology of severe synovitis. However, the number of lumbar lesions with RA might increase because the main pathology is degeneration, not synovitis. Thus, life expectancy should increase, resulting in greater age-related degeneration [9]. The facets and vertebral end-plates are the most important areas affected in the lumbar spine [6,8,10]. Consequently, chronic facet arthritis and enthesopathy at the disco-vertebral junction are important findings in the lumbar spine and are present in up to 25% of patients with RA [1,9]. Common pathological findings in the lumbar spine that are encountered in patients with RA are degenerative spondylolisthesis, endplate erosions, disc space narrowing, facet erosion, facet cysts, and vertebral fractures secondary to osteoporosis [2,11,12]. There have also been reports of rheumatoid nodules causing spinal stenosis and radiculopathy [10,13].

Important causes of revision surgery are implant malposition, inadequate decompression, epidural hematoma, iatrogenic neural injury, and infection. These are commonly encountered in the early post-operative period [14,15]. The causes for delayed reoperation are generally pseudoarthrosis, implant failure, adjacent segment disease (ASD), and, less commonly, late-onset infection [14,15]. In a series assessing the problems of posterior lumbar interbody fusion in the lumbar spine of patients with RA, the authors reported a high rate of complications including collapse of adjacent vertebra, adjacent level instability, and infection [2]. Our study demonstrated an increased overall risk of reoperation in RA patients undergoing lumbar fusion. This effect was more evident 12 months after index surgery. The Kaplan–Meier cumulative event analysis revealed that the reoperation rate was more evident at one year follow-up, and this difference gradually increased until 5 years after the index surgery. The difference stabilized until the final follow-up two years later. ASD is one of the common causes of reoperation after lumbar spinal fusion, especially after 12 months from the index surgery. The rate of ASD in patients with RA has been infrequently described [3,16]. In a study by Crawford et al., the researchers reported similar clinical outcomes in patients with and without RA after lumbar spinal fusion [6]. However, that group studied a small sample of only 19 age-matched cases and controls. The group also reported that the rate of complications was significantly higher in the RA group (37%) than in the controls (21%). Implant failure, osteopenia, and wound infections were frequent concerns in patients with RA undergoing spinal fusion. Most recent advancements in the treatment of RA have centered on the molecular understanding of the autoimmune mechanism and the subsequent development of biologic agents [17,18]. As a consequence, the number of patients reaching end-stage joint destruction has decreased. There has been growing interest in the level of disease activity indicators, such as the 28-joint Disease Activity Score (DAS28) that includes the C-reactive protein (CRP) level (DAS28-CRP), the matrix metalloproteinase 3 (MMP-3) level, and the presence of radiographic ASD. Seki et al. found elevated disease activity indicators (DAS28-CRP and MMP-3) to be associated with radiographic ASD, suggesting that control of disease activity is crucial in preventing ASD after index surgery [5]. Using multivariate logistic regression analysis, these researchers discovered that elevated DAS28-CRP showed the highest positive correlation with ASD incidence after surgery. Therefore, control of disease activity can lead to reduced ASD after surgery. The group recommended beginning biologic therapy before the development of ASD, especially when the patient starts to show early structural changes in weight-bearing joints [5,19]. In their study, 21% of the patients required revision surgery for ASD, and 50% of the patients showed radiographic changes consistent with ASD. This percentage was higher in patients undergoing fusion than in patients undergoing decompression alone. Overall, the rate of revisions in the patients undergoing fusion was 37%, compared to 4% in patients undergoing decompression alone. Of all revision surgeries, 73% were indicated due to symptomatic ASD; ASD was the most frequent reason for revision surgery in their study. Kang et al. reported a significantly higher rate of complications after posterolateral lumbar fusion in RA patients (47.5%) compared to non-RA patients (17.1%). Patients with RA experienced significantly higher rates of ASD (*p* < 0.001). Similarly, in a study by Park et al., patients with RA were associated with a 4.5 times higher risk of fusion-related ASD than patients without RA. Park et al. also concluded that long segment fusion (three-level) was associated with a higher risk of ASD requiring surgery than was a single- or double-level fusion.

Osteoporosis is an important cause of implant failures and vertebral fractures in RA patients undergoing lumbar fusion surgery and increases the likelihood of revision surgeries. Patients with RA have lower bone mineral density than controls [20]. This occurs for two reasons: a chronic inflammatory state and the use of oral corticosteroids in the management of the disease [21]. A few authors have suggested a positive correlation between oral corticosteroid use and the risk of osteoporotic fractures, but others have found either no correlation or an inverse relationship [20,22,23,24]. In patients with osteoporosis, starting appropriate anti-resorptive or anabolic medications to prevent associated implant-related complications and vertebral fractures is of prime importance. Our study demonstrated a higher overall risk of reoperation in the RA group with osteoporosis, and this effect was more pronounced within 3 months of index surgery. This seems to be associated with early implant failure due to poor bone quality.

The major limitations of most previous studies on this topic are their small sample sizes and single-center experiences. There has been nationwide analysis of RA in cervical spine fusions but not lumbar spine fusions [25]. Our current study, based on a nationwide database, has provided insight on this topic. There are some important limitations to our study. The most important is that the level of disease activity might not have been uniform in all the patients included in the study; the medical management methods also varied by institution. Additionally, the exact reasons for revision surgery in the patients undergoing reoperation were unknown.

However, we analyzed the risk of reoperation following lumbar spinal fusion according to the time point. As many existing studies have reported the typical causes of reoperation following lumbar spinal fusion by time point, we can infer the cause of reoperation. Another limitation is that the choice of surgery was controlled by individual surgeons whose experiences and recommendations vary. This is difficult, if not impossible, to control in a population-based study.

## 5. Conclusions

This population-based cohort study showed that patients with RA had a 1.31 times higher risk of reoperation than controls. The Kaplan–Meier cumulative event analysis demonstrated that a higher risk of reoperation in the RA group occurred 1 year post-surgery. This risk increased further until 5 years post-surgery and was then stable through to the last follow-up at 7 years.

## Figures and Tables

**Table 1:** Comparison between the demographic characteristics and comorbidities of rheumatoid arthritis (RA) patients and the control group.

	RA Group (*n* = 2239)	Control Group (*n* = 11,195)	Total (*n* = 13,434)	*p*-Value
** Age **	64.5 ± 8.8	64.5 ± 8.8	64.5 ± 8.8	0.96
** Sex (male) **	603 (26.9%)	3033 (27.1%)	3636 (27.1%)	0.876
** Age group **				
40–49	112 (5.0%)	561 (5.0%)	673 (5.0%)	0.997
50–59	562 (25.1%)	2785 (24.9%)	3347 (24.9%)	
60–69	839 (37.5%)	4207 (37.6%)	5046 (37.6%)	
≥70	726 (32.4%)	3642 (32.5%)	4368 (32.5%)	
** Comorbidity **				
DM	594 (26.5%)	2710 (24.2%)	3304 (24.6%)	0.02
Depression	308 (13.8%)	951 (8.5%)	1259 (9.4%)	<0.001
Osteoporosis	662 (29.6%)	2432 (21.7%)	3094 (23.0%)	<0.001
Parkinson	25 (1.1%)	120 (1.1%)	145 (1.1%)	0.852
Peripheral vascular	371 (16.6%)	1580 (14.1%)	1951 (14.5%)	0.003
ESRD	20 (0.9%)	99 (0.9%)	119 (0.9%)	0.967
Liver disease	279 (12.5%)	929 (8.3%)	1208 (9.0%)	<0.001
HTN	1305 (58.3%)	5986 (53.5%)	7291 (54.3%)	<0.001
Cerebrovascular	224 (10.0%)	1029 (9.2%)	1253 (9.3%)	0.227
Chronic pulmonary	560 (25.0%)	2110 (18.8%)	2670 (19.9%)	<0.001
** Comorbidity ** ** number **				
0	596 (26.6%)	3781 (33.8%)	4377 (32.6%)	<0.001
1–2	1393 (62.2%)	6535 (58.4%)	7928 (59.0%)	
≥3	250 (11.2%)	879 (7.9%)	1129 (8.4%)	

**Table 2:** Crude and adjusted hazard ratios for reoperation in rheumatoid arthritis (RA) patients compared with the control group.

Patient Group	*n*	Reoperation	Duration	Rate	HR (95% CI)	
					Crude HR	Adjusted HR
**Controls**	**11,195**	**958**	**1032.2 ± 700.9**	8.8	1 (ref.)	1 (ref.)
**RA patients**	2239	265	964.8 ± 647.2	11.8	1.44 (1.24–1.66)	1.31 (1.10–1.60)

**Table 3:** Adjusted hazard ratios of reoperation in rheumatoid arthritis (RA) patients comparedwith the control group based on the time between initial operation and reoperation.

<3 Months				3 Months–1 Year				>1 Year			
Reoperation	Rate	Adjusted HR (95% CI)	*p*-Value	Reoperation	Rate	Adjusted HR (95% CI)	*p*-Value	Reoperation	Rate	Adjusted HR (95% CI)	*p*-Value
77	0.69	1 (ref.)	0.699	168	1.50	1 (ref.)	0.44	740	6.61	1 (ref.)	0.002
15	0.67	1.13 (0.61–2.11)		42	1.88	0.87 (0.61–1.24)		208	9.29	1.31 (1.11–1.57)	

**Table 4:** Subgroup analysis of comorbidity factors related to the risk of reoperation in RA patients.

Variables		<3 Months		3 Months–1 Year		<1 Year	
		Adjusted HR (95% CI)	*p*-Value	Adjusted HR (95% CI)	*p*-Value	Adjusted HR (95% CI)	*p*-Value
Sex	Female	1 (ref.)		1 (ref.)		1 (ref.)	
	Male	1.04 (0.67–1.66)	0.860	0.70 (0.50–0.97)	0.031	1.12 (0.96–1.32)	0.153
Age group	40–49	1 (ref.)		1 (ref.)		1 (ref.)	
	50–59	1.18 (0.42–3.35)	0.750	0.52 (0.24–1.17)	0.114	0.69 (0.47–1.03)	0.072
	60–69	1.76 (0.65–4.76)	0.266	0.64 (0.30–1.35)	0.239	0.74 (0.50–1.09)	0.123
	≥70	1.00 (0.35–2.83)	0.997	0.85 (0.40–1.81)	0.677	0.86 (0.58–1.29)	0.470
Osteoporosis	No	1 (ref.)		1 (ref.)		1 (ref.)	
	Yes	2.14 (1.15–4.02)	0.017	1.02 (0.70–1.49)	0.914	1.04 (0.87–1.23)	0.698
Parkinson’s	No	1 (ref.)		1 (ref.)		1 (ref.)	
	Yes	1.64 (0.20–13.85)	0.647	1.54 (0.46–5.21)	0.486	1.15 (0.66–2.00)	0.621
DM	No	1 (ref.)		1 (ref.)		1 (ref.)	
	Yes	1.42 (0.82–2.43)	0.209	0.82 (0.59–1.13)	0.225	1.05 (0.89–1.24)	0.571
Depression	No	1 (ref.)		1 (ref.)		1 (ref.)	
	Yes	2.38 (1.09–5.19)	0.030	1.37 (0.92–2.05)	0.125	0.94 (0.74–1.19)	0.595
Comorbidity	0	1 (ref.)		1 (ref.)		1 (ref.)	
	1–2	0.67 (0.37–1.21)	0.184	0.97 (0.68–1.39)	0.881	0.93 (0.78–1.10)	0.394
	≥3	0.48 (0.19–1.21)	0.118	0.98 (0.59–1.64)	0.949	0.90 (0.68–1.20)	0.475

## Data Availability

The present study analyzed the NHI claims data in South Korea. The authors do not have any special access privileges to these data. The data of the NHI claims are accessible to researchers with permission of the HIRA in South Korea. Qualified, interested researchers may request access to these data from the HIRA (http://opendata.hira.or.kr/home.do (accessed on 5 March 2021).
